# Decoding the Structural Bases of D76N ß2-Microglobulin High Amyloidogenicity through Crystallography and Asn-Scan Mutagenesis

**DOI:** 10.1371/journal.pone.0144061

**Published:** 2015-12-01

**Authors:** Matteo de Rosa, Alberto Barbiroli, Sofia Giorgetti, Patrizia P. Mangione, Martino Bolognesi, Stefano Ricagno

**Affiliations:** 1 Dipartimento di Bioscienze, Università di Milano, Via Celoria 26, 20133, Milano, Italy; 2 Dipartimento di Scienze per gli Alimenti, la Nutrizione e l’Ambiente, Università di Milano, Via Celoria 2, 20133, Milano, Italy; 3 Dipartimento di Medicina Molecolare, Istituto di Biochimica “A. Castellani”, Università di Pavia, Via Taramelli 3/b, 27100, Pavia, Italy; 4 CIMAINA and CNR-Istituto di Biofisica, c/o Università di Milano, Via Celoria 26, 20133, Milano, Italy; Universitat Autònoma de Barcelona, SPAIN

## Abstract

D76N is the first natural variant of human β-2 microglobulin (β2m) so far identified. Contrary to the wt protein, this mutant readily forms amyloid fibres in physiological conditions, leading to a systemic and severe amyloidosis. Although the Asp76Asn mutant has been extensively characterized, the molecular bases of its instability and aggregation propensity remain elusive. In this work all Asp residues of human β2m were individually substituted to Asn; D-to-N mutants (D34N, D38N, D53N, D59N, D96N and D98N) were characterised in terms of thermodynamic stability and aggregation propensity. Moreover, crystal structures of the D38N, D53N, D59N and D98N variants were solved at high-resolution (1.24–1.70 Å). Despite showing some significant variations in their thermal stabilities, none showed the dramatic drop in melting temperature (relative to the wt protein) as observed for the pathogenic mutant. Consistently, none of the variants here described displayed any increase in aggregation propensity under the experimental conditions tested. The crystal structures confirmed that D-to-N mutations are generally well tolerated, and lead only to minor reorganization of the side chains in close proximity of the mutated residue. D38N is the only exception, where backbone readjustments and a redistribution of the surface electrostatic charges are observed. Overall, our results suggest that neither removing negative charges at sites 34, 38, 53, 59, 96 and 98, nor the difference in β2m pI, are the cause of the aggressive phenotype observed in D76N. We propose that the dramatic effects of the D76N natural mutation must be linked to effects related to the crucial location of this residue within the β2m fold.

## Introduction

**Fig 1:**
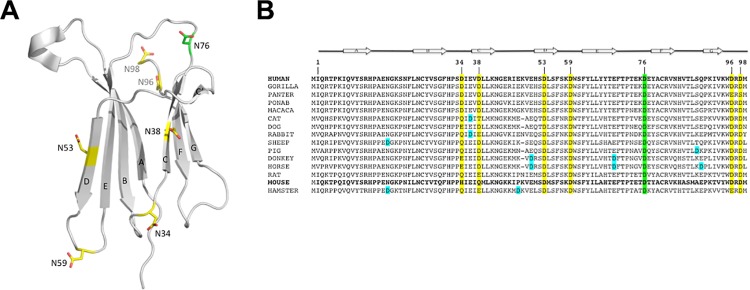
Distribution and conservation of Asp residues in β2m. (A) Asp residues are mapped on a ribbon representation of wt β2m structure (pdb code 2YXF), and shown as stick models. (B) Alignment of mammalian β2m amino acid sequences, numbering according to the mature human protein; D76 is highlighted in green, while other conserved Asp residues are highlighted in yellow. Asp residues randomly located in different sequences are shown in cyan.

Beta-2 microglobulin (β2m) is a 99-residue protein, physiologically acting as the light chain of the major histocompatibility complex (MHC) class I [1]. β2m displays a classic immunoglobulin fold, and is well conserved among vertebrates. β2m tertiary structure consists of a seven-stranded beta-sandwich; according to standard nomenclature, β-strands are named from A to G, and loops are named after the neighbouring β-strands (Fig 1A). The characterization of β2m structure and folding dynamics gained considerable resonance when the protein was found to be responsible for Dialysis Related Amyloidosis (DRA) [2, 3]. In 2012 Valleix *et al*. reported the first β2m natural mutant, bearing the D76N mutation. The mutant protein is the etiological agent of a previously unknown systemic amyloidosis, whereby patients carrying the D76N mutation accumulate large mutant β2m amyloid deposits, especially in internal organs. Opposite to what is observed in DRA, the D76N β2m mutant does not accumulate at high concentration in patients’ sera [4]. Moreover, it displays *in vitro* a striking tendency to aggregation and, contrary to wild type (wt) β2m, the D76N mutant aggregates under non-denaturing unseeded conditions [4, 5]. Particularly, high aggregation levels can be reached *in vitro* under agitation in the presence of hydrophobic surfaces, *i*.*e*. under conditions that the D76N mutant experiences in the interstitial fluid where it aggregates [3, 5].

The D76N mutant is thermodynamically much less stable compared to wt β2m, displaying markedly decreased melting temperature (Tm) and melting concentration (Cm) values in thermal and chemical unfolding experiments, respectively [4–6]. Although the D76N mutant is less stable than wt β2m and very aggregation prone, it escapes the protein quality control system, which in the endoplasmic reticulum targets and degrades unfolded or aggregated proteins [4, 6]. We previously showed that the D76N mutant is efficiently stabilised when assembled in the MHC class I complex: in fact, MHC class I complexes hosting either the D76N mutant, or the wt β2m, show the same 3D structure, fold stability, and dynamics. Therefore, MHC class I has been proposed to prevent aggregation, stabilising the D76N mutant as long as it is part of the complex [6].

From the structural point of view, the D76 residue lies in the β2m EF loop and it is completely solvent exposed. The crystal structure of the D76N mutant matches very closely that of wt β2m, however, in this mutant, the H-bond network in the protein EF-loop is reorganised, leading to a more rigid EF-loop, as shown by analysis of the crystallographic B-factors [4]. In keeping with the crystallographic data, NMR analysis of the D76N mutant does not reveal any major structural difference compared with the wt protein in solution [5], confirming the modest overall impact of the D76N mutation on the β2m 3D structure.

In search of an explanation for the remarkable D76N mutant instability and aggregation propensity, we focussed on the effects of the negative charge loss coupled to the D76N mutation. In this respect, we considered that one (or more) of the six β2m Asp residues (other than D76) might display similar effects, once replaced with N, since any D-to-N mutation would result in an increase of β2m theoretical isoelectric point (pI) from 6.07 to 6.46. Notably, protein molecules are more likely to aggregate at a pH close to their pI due to decreased coulombian repulsion; hence, a pI change might be a component of the observed D76N increased aggregation propensity. In human β2m the seven Asp residues fall at sites 34, 38, 53, 59, 76, 96 and 98, and their conservation is high throughout the mammalian β2m sequences (Fig 1B). The Asp residues are evenly located over the β2m fold, in stretches of secondary structure (D38 and D53), loops (D34, D59 and D76) and in the C-terminal tail (D96 and D98) (Fig 1A).

To validate the above hypothesis we systematically mutated all Asp residues to N (D-to-N mutations), and isolated the corresponding single-site mutants, hereafter named D34N, D38N, D53N, D59N, D96N and D98N. The mutants were biochemically and structurally characterised for their secondary structure content and fold stabilities, for their aggregation propensities, and the crystal structures of D38N, D53N, D59N and D98N mutants were determined. The data on protein stability and aggregation propensity indicate that all the D-to-N mutants more closely match the wt β2m properties rather than the D76N variant’s. The structural analyses show that the different D-to-N mutations affect differently the β2m fold, which is however only moderately perturbed.

Based on the results here reported, we speculate that the decreased stability and remarkable aggregation trends of the D76N β2m mutant must be the result of specific yet uncharacterized properties, strictly linked to the structural location of the protein 76 site.

## Methods

### Mutagenesis, expression and purification

β2m D to N mutants (D34N, D38N, D53N, D59N, D96N, D98N) were produced using the phusion site-directed mutagenesis kit (Lifetechnologies), following the manufacturer protocol. Expression and purification of monomeric wt and β2m variants were carried out as described previously [7].

### Aggregation propensity

Samples of recombinant variants D34N, D38N, D53N, D59N, D76N, D96N and D98N and wild type β2m, 100 μL at 40μM in PBS (pH 7.4) containing 10 μM Thioflavin T (ThT) (SIGMA)**, were incubated at 37°C in Costar 96-well black-wall plates sealed with clear sealing film (4TITUDE) and were subjected to 900 rpm double-orbital shaking. Bottom fluorescence was recorded at 15-min intervals (BMG LABTECH FLUOstar Omega). Fluorescence was monitored in three or more replicate tests.

### Circular dichroism

**Fig 2:**
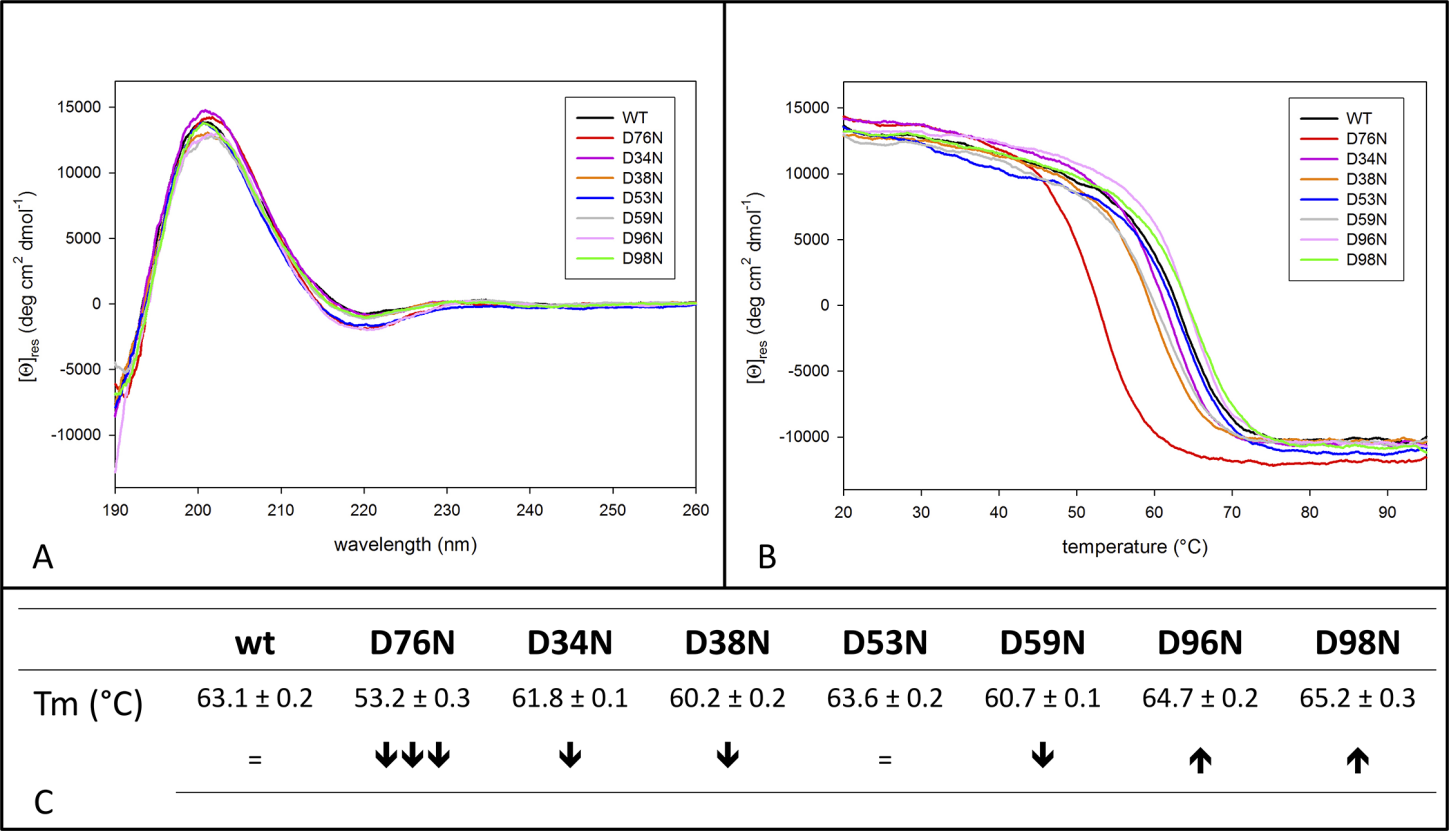
Thermodynamic stability of β2m D-to-N variants. (A) Comparison of the CD spectra of wt β2m and D-to-N variants. (B) Thermal stability assessed by CD experiments in the far-UV region. (C) Tm values are determined as the minima of the first derivative of the unfolding profiles, each thermal unfolding was repeated four times. Standard deviations were calculated and are shown for each Tm. Arrows highlight graphically the Tm differences.

Far-UV spectra (190–260 nm) and thermal unfolding (monitored at 202 nm in the 20–95°C temperature range) for β2m D34N, D38N, D53N, D59N, D96N, D98N were measured using a JASCO J-810 spectropolarimeter equipped with a Peltier device and a fluorescence detector (JASCO corporation, Tokyo, Japan). All temperature ramps were performed in 50 mM sodium phosphate, pH 7.4, if not otherwise stated; the protein concentration was 0.1 mg/mL (cell path 0.1 cm), and the ramp slope was set at 50°C/h. Tm values were determined as the minima of the first derivative of the unfolding profiles. Thermal unfolding experiments were repeated four times for each β2m variant: the Tms shown in Fig 2C represent the average values from the four experiments, the corresponding standard deviations were also calculated.

### Crystallisation, structure solution and analysis

Crystallisation screens were set up for all six D-to-N mutants, and four of them, *i*.*e*. D38N, D53N, D59N, and D98N were successfully crystallized. The conditions are the ones commonly used for most of the β2m monomeric variants so far characterized: 21–27% PEG 4000, 15% glycerol, 0.2 M ammonium acetate, 0.1 M sodium acetate, pH 5.0–5.5. Single crystals were flash-frozen in liquid nitrogen without additional cryoprotectants, and X-ray diffraction data were collected at beamline ID23-1 (ESRF, Grenoble) at 100 K. Data were processed with MOSFLM and SCALA [8, 9] and structures determined by molecular replacement using the program PHASER [10] and monomeric wt β2m as the search model (pdb ID 2YXF). All the structures were refined with phenix refine [11]; manual model building performed with COOT [12]. Analysis of the structures (calculation of RMSD values and surface electrostatic potential, identification of H-bonds/polar contacts) was performed with Pymol (The PyMOL Molecular Graph- ics System; Schrodinger, LLC, Portland, OR, USA), using default parameters. Pymol was also used for the preparation of the figures. Average B-factors for the main chain atoms extracted from Baverage [8] were scaled according to the following equation: B_z-score(i)_ = (Bx_(i)_ - 〈B〉_(i)_)/σ_(i)_

Where Bx_(i)_ is the B-factor for residue x in the *i* structure, 〈B〉_(i)_ is the arithmetical average of the B-factors in the *i* structure, and σ_(i)_ the corresponding standard deviation.

### Structure deposition

Atomic coordinates and structure factors for the β2m D38N, D53N, D59N, and D98N mutants were deposited in the Protein Data Bank, with the ID codes 4RMR, 4RMS, 4RMQ and 4RMT, respectively.

## Results

### Distribution and conservation of aspartate residues in mammalian β2m

β2m sequences display a high degree of amino acid conservation in mammals (Fig 1B). Among the orthologs used in this analysis, sequence identity varies between 66% (dog *versus* hamster β2m) and nearly 100% (among primates). The human protein harbours seven Asp residues, five of them (D53, D59, D76, D96 and D98) are almost 100% conserved in all the sequences analysed, while D34 and D38 are found substituted mostly by E or by other polar-uncharged amino acids. All seven Asp residues are solvent exposed, however the charge distribution around them varies considerably. D38 and D76 negative charges are surrounded by a number of positive charges: R45 and R81 establish salt bridges with D38, while K41 and K75 are in close vicinity of D76. Conversely, D96 and D98 fall in a negatively charged region, clustering with E74 and E77. The remaining three Asp residues (D34, D53 and D59) are located in regions of lower/mixed surface charge.

Although the number of Asp residues, and in general of negatively charged amino acids, is relatively conserved, mammalian β2m sequences display a wide range of pIs, ranging from 5.7 to 8 (Table 1). The human protein is one of the most acidic (pI = 6.07), and the D76N mutation increases such value by 0.5 units. Murine β2m, whose sequence is 70% identical to the human β2m, is not amyloidogenic, and has been used as an *in vitro* inhibitor of human β2m aggregation [13, 14]. Relative to human β2m, murine β2m is characterized by lower thermal stability and higher pI (7.97), suggesting that these parameters do not necessarily correlate with the aggregation propensity.

**Table 1:** Theoretical isoelectric points for mammalian wt β2m’s and the human D76N variant. The number of D (E) residues is also reported.

	pI	n° D (E)
**human WT**	6.07	7 (8)
**human D76N**	6.46	6 (8)
**mouse**	7.97	5 (6)
**rat**	7.08	5 (7)
**dog**	5.67	6 (9)
**cat**	5.91	6 (7)
**hamster**	7.08	6 (7)
**horse**	6.45	9 (5)
**donkey**	6.45	8 (6)
**pig**	7.95	7 (5)
**rabbit**	6.06	7 (7)
**macaque**	6.46	7 (7)
**gorilla**	6.07	7 (8)
**chimpanzee**	6.46	5 (9)
**orangutan**	6.46	7 (7)

### Thermal stability and aggregation propensity of β2m mutants

In order to compare the solution secondary structure content and the protein conformation of all D-to-N mutants with D76N and wt β2m, the far-UV circular dichroism (CD) spectra of all the variants were recorded. Fig 2A shows that the resulting spectra are essentially identical, indicating a high structural conservation for all the variants, in solution and under the conditions tested.

To assess the conformational stability of D34N, D38N, D53N, D59N D96N and D98N mutants, thermal unfolding was monitored by CD in the far-UV region, using both wt and D76N variants as controls. None of the variants showed a marked Tm variation (relative to wt β2m), however some features are worth noting (Fig 2B and 2C). Both D96N and D98N mutants melt with a Tm 1.3°C higher than wt β2m. Although such ΔTm is relatively small, the consistency of the two values (for two mutants that concern nearby residues) adds significance and indicates that removal of a negative charge from the C-*terminal* region slightly stabilises the β2m fold. While D53N shows exactly same Tm of the wt protein, the Tm drop for D34N, D38N and D59N (whose values are 1.8, 3.4 and 2.8°C lower than wt β2m, respectively) is observable. Given the contained standard deviations calculated for the measured Tm values (Fig 2C), also ΔTm as small as 1.3°C can be considered significant.

**Fig 3:**
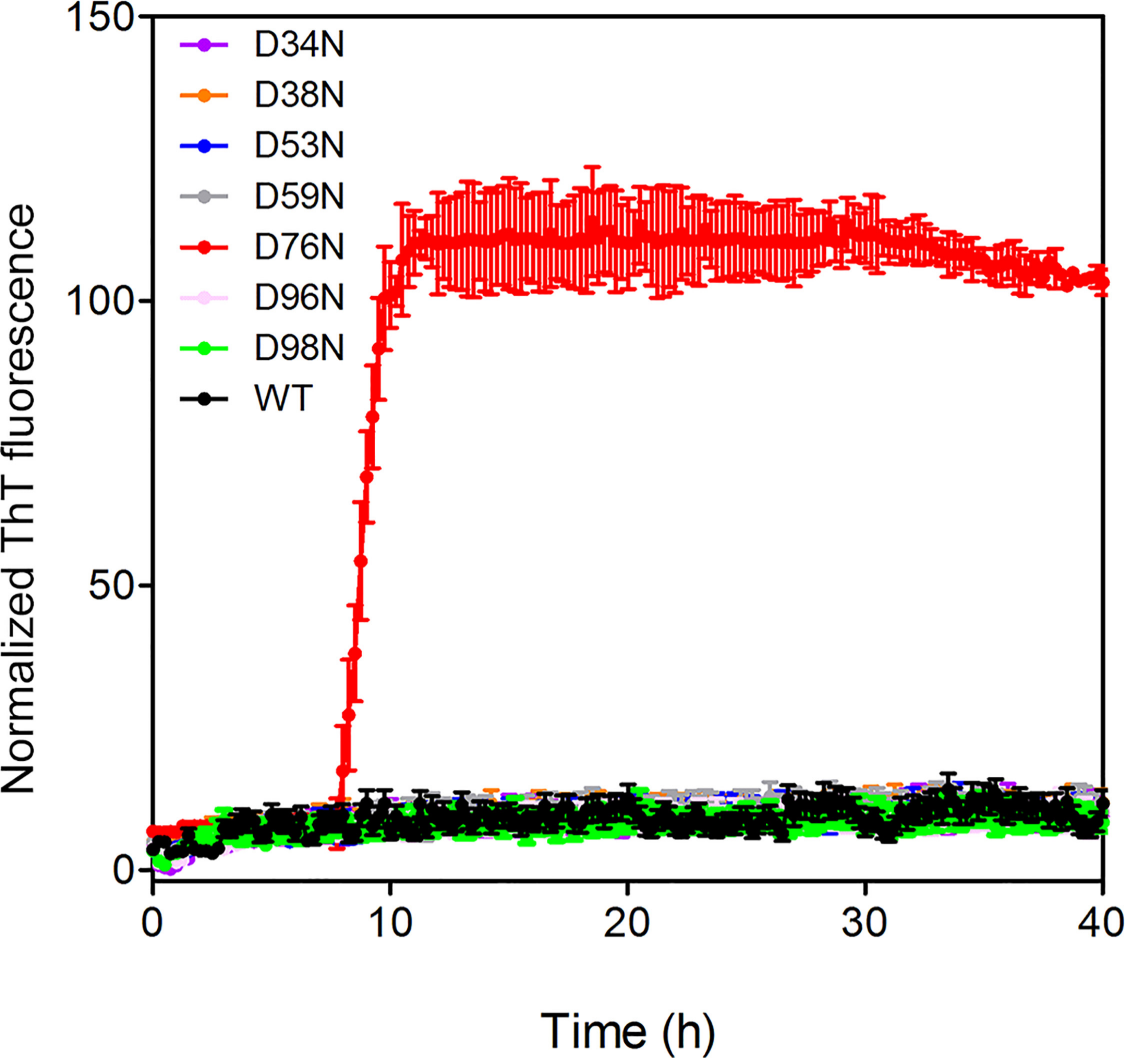
Aggregation of wt β2m and of D-to-N variants. Under shaking conditions in physiological buffer, an increase of ThT signal was recorded solely for D76N β2m (red), no signal increase was registered for the wt protein (black) or the other variants, whose signals are comprised in the base line: D34N (brown), D38N (orange), D53N (blue), D59N (grey), D96N (pink), D98N (green).

In order to verify the aggregation propensities of the D-to-N engineered variants, we performed experiments at physiological pH with mild shaking. Under such conditions, as previously described, the D76N variant efficiently forms fibrils within few hours, while wt β2m remains natively folded and soluble [5]. Fig 3 shows that the control D76N β2m undergoes abundant aggregation in the first 10 hours, while for wt β2m and all other six D-to-N variants there is no increase in ThT signal within the time frame of the experiment. As a further control on the most destabilized mutant (D38N), turbidity measurements were performed during the course of the aggregation experiments: the solution remained clear within the time frame of the experiment. Native protein gel indicates that at the end of incubation the protein is monomeric and indistinguishable from the native protein used as a control (data not shown).

### Crystal structures of D38N, D53N, D59N and D98N β2m

Crystals of D38N, D53N, D59N and D98N β2m readily appeared under crystallization conditions similar to those used for wt and other monomeric β2m proteins. The crystal structures of these variants were determined and refined at high resolution (1.53, 1.70, 1.46 and 1.24Å, for D38N, D53N, D59N and D98N, respectively) with quite satisfactory R_work_ and R_free_ values (Table 2). All the β2m residues could be traced for the four crystallized variants, thanks to the high resolution achieved and the quality of the electron density maps; only the side-chains of M99 in D38N, and K75 in D98N, could not be modelled. Notably, the D98N variant 3D-structure is among ones at the highest resolution for a monomeric β2m mutant.

**Table 2:** Data collection and refinement statistics.

	D38N	D53N	D59N	D98N
**Data collection**				
Space group	I 1 2 1	I 1 2 1	I 1 2 1	I 1 2 1
Unit cell parameters	53.3, 29.1, 71.2;	55.0, 28.9, 67.2;	54.8, 28.9, 67.2;	54.8, 29.0, 67.6;
a, b, c (Å); α, β, γ (°)	90.0, 92.0, 90.0	90.0, 101.9, 90.0	90.0, 101.8, 90.0	90.0, 102.0, 90.0
Unique reflections	16,017	10,917	17,472	28,992
Resolution range (Å)	26.16–1.53 (1.56–1.53)	26.45–1.70 (1.73–1.70)	23.24–1.46 (1.49–1.46)	19.05–1.24 (1.26–1.24)
I/σ(I)	8.4 (2.1)	5.4 (2.1)	10.5 (3.6)	10.4 (2.2)
Completeness (%)	97.4 (98.5)	94.5 (92.1)	96.3 (96.0)	98.1 (98.7)
Multiplicity	3.5	2.9	2.9	4.4
**Refinement**				
Resolution range (Å)	26.15–1.53	26.45–1.70	23.24–1.46	19.05–1.24
R_work_ / R_free_ ^*^ (%)	19.4/24.7	19.0/24.9	16.6/19.2	16.2/ 19.8
RMSD				
Bonds (Å)	0.018	0.008	0.008	0.013
Angles (°)	1.737	1.133	1.198	1.473
Ramachandran plot				
In preferred regions (%)	98	97	96	97
In allowed regions (%)	2	3	4	3
Outliers (%)	0	0	0	0
B-factors (Å^2^)^#^	34	27	21	21

Values in parentheses refer to the highest resolution shells.

*Rwork = Σhkl||Fo|—|Fc|| ⁄ Σhkl|Fo| for all data, except 10%, which were used for Rfree calculation.

^#^Average temperature factors over the whole structure.

The D53N, D59N and D98N 3D-structures superimpose well with both wt β2m and the D76N mutant (RMSD values in the 0.10–0.17 and 0.63–0.73 Å range, respectively) (Table 3); most of the backbone structural differences, relative to wt β2m, are located in the protein region comprising loops AB, EF, CC’D and the C-terminal tail. The RMSD values suggest that the three variants match more closely the wt β2m structure than that of the highly amyloidogenic D76N mutant.

**Table 3:** RMSD values (Å) for the 3D structural comparisons of the D-to-N mutants with wt and D76N β2m (pdb code 2YXF and 4FXL, respectively). The number of Cα atoms used for each calculation is reported in parenthesis.

	wt	D76N
wt	-	0.60 (97)
D76N	0.60 (97)	-
D38N	3.44 (97) 1.12 (89)*	3.44 (97) 1.29 (89)*
D53N	0.17 (97)	0.71 (97)
D59N	0.10 (97)	0.70 (97)
D98N	0.15 (97)	0.63 (97)

* Atoms belonging to AB loops were excluded in this calculation

**Fig 4:**
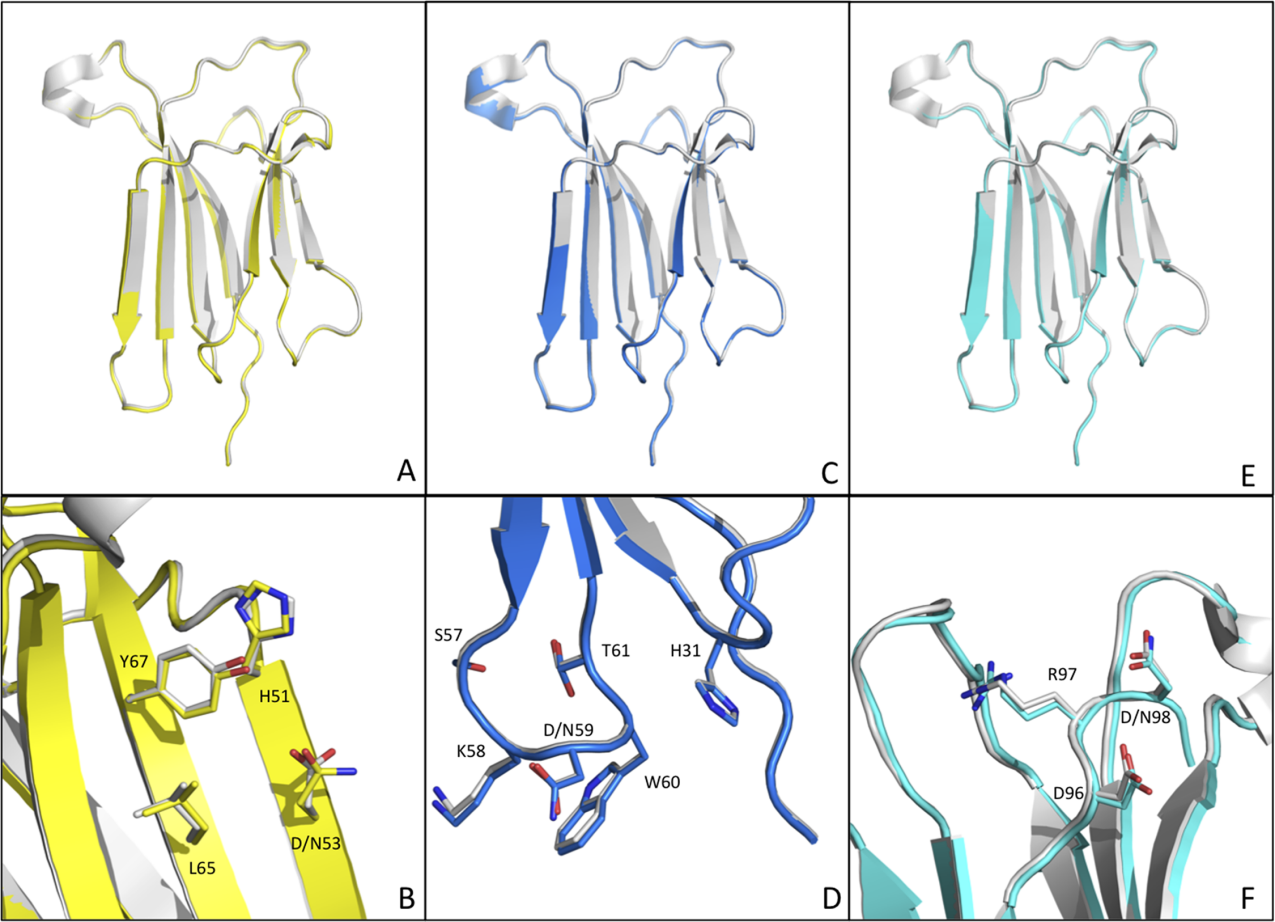
Comparison of the crystal structures of D-to-N β2m variants. (A), (C) and (E) Cartoon representation of the β2m variants D53N (yellow), D59N (blue) and D98N (cyan) individually overlaid onto the structure of wt β2m (light grey, PDB ID 2YXF). (B), (D) and (F) Close up views of the mutation sites in D53N, D59N and D98N, color coded as in A C and E panels; side chains of the mutated and neighboring residues are shown as sticks and labeled.

In detail, residue D53 lies in the middle of β2m D-strand, one of the edge strands of the four-stranded β-sheet (strands ABED). D53 is involved in the interaction with the MHC class I heavy chain; however, in the isolated β2m, it is totally solvent exposed and devoid of interactions with neighbouring residues (Fig 4B). The D53-to-N substitution does not alter the regularity of the D-strand, as both D53 and N53 share the same region of the Ramachandran plot (Phi/Psi angles of residues 53 are -109°/113°, and -129°/98° for wt β2m and the D53N variant, respectively). D53 is the only wt β2m aggregation-protective element (namely an inward-pointing charge) at that edge of the four-stranded β-sheet, according to the analysis by Richardson and Richardson [15]. However, even if the D-to-N mutation leaves a regular β-strand, unprotected and potentially prone to aggregation, the D53N mutant does not show any increased aggregation propensity.

Residue D59 falls in the DE-loop, which is a hot spot for β2m stability and aggregation [16, 17], its side chain is not involved in any interaction with adjacent residues. Given that β2m thermodynamic stability and aggregation propensity strongly depend on DE-loop backbone geometry [16], expectedly the side chain substitution (D to N) does not affect β2m fold stability. Among the DE loop mutants previously studied [16, 17], D59N is the first variant where only the charge of the DE-loop has been altered. Thus, the DE-loop appears to withstand removal of a negative charge without sizeable effects on β2m structure and stability (Fig 4C, Table 3).

Although D98N is the highest resolution structure (1.24 Å) here presented, the electron density in the area surrounding the mutation is not of excellent quality as elsewhere in the 3D structure. The side chains of the C-terminal residues (R97, N98 and M99) and of the spatially neighbouring E74 and E77 (in the EF-loop) are only partially visible in the electron density. Moreover, the electron density of the EF-loop is weak between residues 75–76, suggesting transmission of conformational flexibility from the C-*terminus* to the EF-loop region.

**Fig 5:**
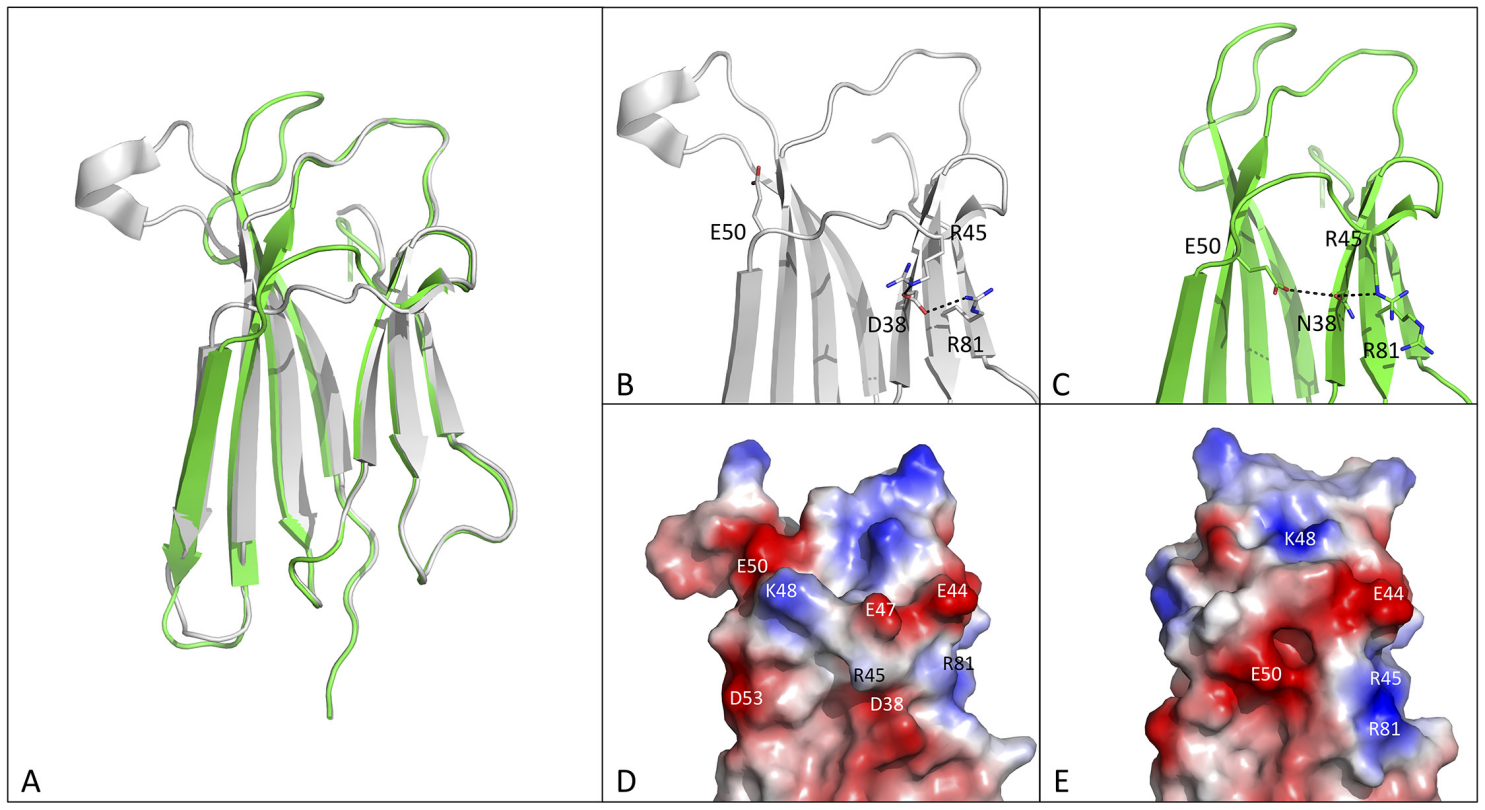
Conformational changes in the D38N mutant. (A) Superposition of wt β2m (light grey, PDB ID 2YXF) on the D38N mutant (green). Close up view of the mutation site in the D38N β2m mutant (C) compared with the wt protein (B). Surface electrostatic charge in the D38N mutant (E) compared with the wt protein (D); visible area and protein orientation as in panels B and C (residues contributing surface charges are labelled).

Contrary to the D53N, D59N and D98N mutants, the structure of the D38N mutant displays evident conformational changes relative to wt β2m and the other D-to-N variants (Fig 5). RMSD values calculated between D38N and both wt β2m and D76N are over 3 Å, when 97 Cα atoms are taken in account (Table 3). The values drop to about 1.2 Å when the β2m AB-loop, which is found in the closed conformation in D38N, is excluded from the calculation (Table 3). Typically, the AB-loop adopts the closed conformation when β2m is part of the MHC class I complex, but such conformation has also been observed in β2m chains separated from the MHC class I complex).

The β2m region surrounding the D38N mutation site undergoes significant conformational changes, leading to rearrangements in both the CC’D-loop and the C-terminal tail (Fig 5). Moreover, in D38N variant both D and E strands are longer due to torsion of Psi angle of S57 and the formation of a H-bond between the O atom of this residue and the N of S61. In the wt β2m structure, residue D38 resides in an area rich in positively charged residues, and establishes H-bonds with both R45 and R81 (Fig 5). Upon D38N mutation, the interaction with R81 is lost, while H-bonding is maintained with R45. To compensate for the lack of the D38 negative charge, the E50 side chain moves toward the mutation site, bringing the E50 carboxylate at H-bonding distance with N38 and, in parallel, pulling along all the CC’D-loop. Overall, we observe a charge redistribution in the region formed by the edge strands C and D (Fig 5).

### B-factors analysis

**Fig 6:**
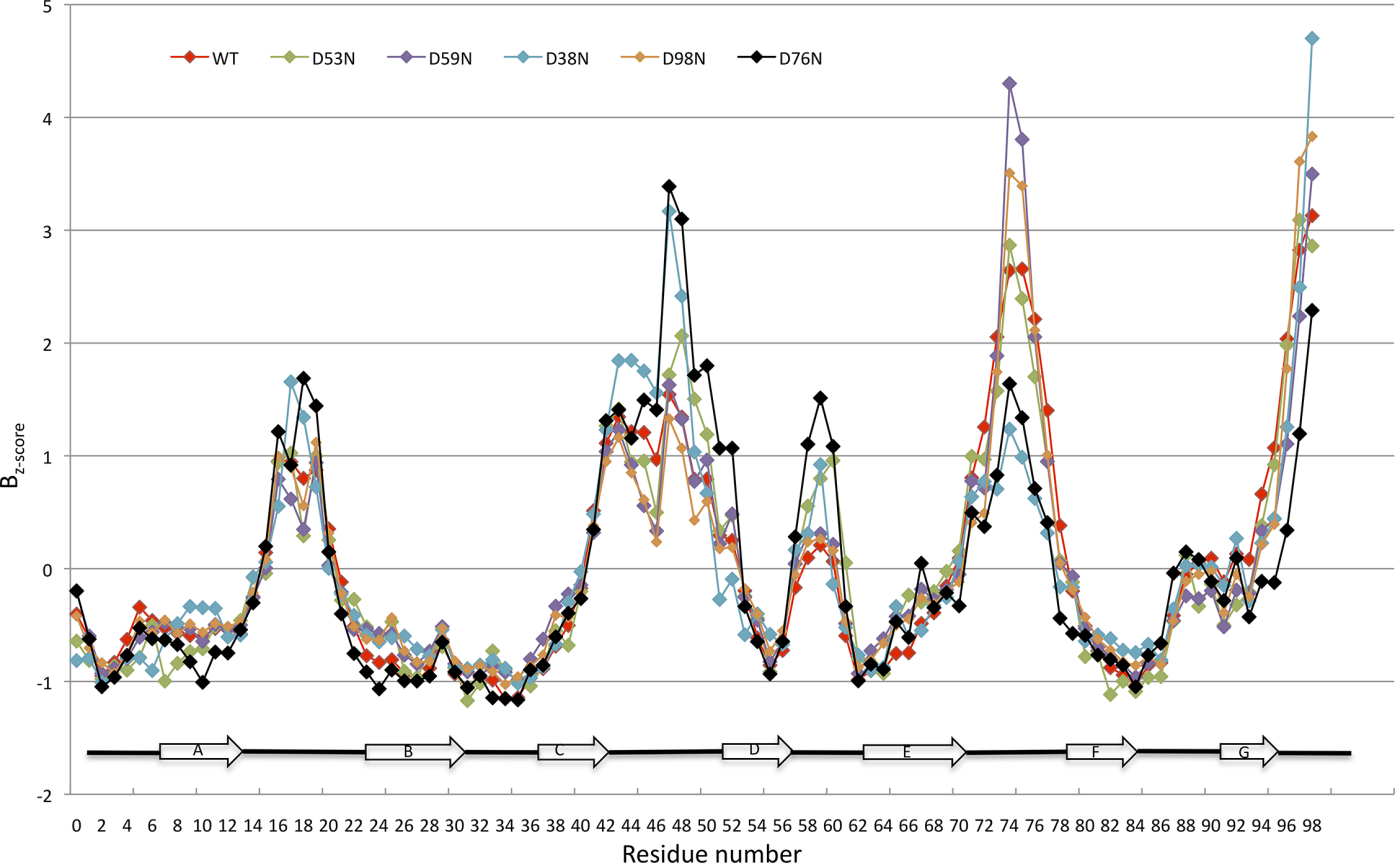
Comparison of the B_z-score_ profiles for wt β2m and four D-to-N mutants. Plot of the B_z-score_ *versus* the residue number. Data for wt β2m (red, pdb ID 2YXF), D76N (black, pdb ID 4FXL), D38N (cyan), D53N (green), D59N (purple), and D98N (orange) are presented. β-strands building up the β2m fold are shown as arrows, and labelled in the lower part of the graph.

As reported above, the four crystallised variants show small but significant differences in terms of thermal stability. Taking advantage of the high resolution crystal structures, we analysed the distribution of B-factors along the β2m polypeptide chain, as in this resolution range (<1.9 Å) such parameters well correlate with the protein local dynamic behaviour [18]. In order to remove any bias due to differences in crystal quality and refinement protocols, B-factors (averaged over each residue backbone atoms) were scaled and the B_z-score_ parameter (see Materials and Methods) was used for the comparisons (Fig 6). The D53N, D59N and D98N variants, which match very closely the wt 3D structure, show a similar B_z-score_ pattern, with positive peaks corresponding to AB-, CC’D-, DE-, EF-loops, and the C-*terminus*. Differences of roughly 1 or more standard deviations with respect to the wt structure can be observed only in the in the DE-loop of the D53N mutant, and in the EF-loop of D59N and D98N. Interestingly, none of the mutations leads to an increase of the B_z-score_ in the polypeptide segments where they are located, but rather affects structurally adjacent loops. The analysis of D38N B_z-score_ distribution shows a pattern similar to the D76N variant: the EF-loop (hosting N76 in the pathologic mutant), is more rigid than in all other variants, while the CC’D-loop (as in D76N) and the C-terminal tail show higher flexibility. Surprisingly, the AB-loop, although found in the closed conformation, displays local B_z-score_ comparable to those of the other variants.

## Discussion

D76N is the first natural variant of β2m, and the only one so far discovered [4]. No other isoforms or variants due to single nucleotide polymorphism or alternative splicing are known. The only other mutations identified in the human β2m gene fall in the region coding for the propeptide, or create frameshifts, in either cases affecting β2m circulating levels rather than translating into a protein with altered properties [19, 20]. The β2m found in the ultrafiltrate of DRA patients showing Asp instead of an Asn at position 17 was due to posttranslational deamination of the residue, given that the sequence of the corresponding β2m gene was wild type [21]. Lack of reported mutations, and sequence conservation that is high among all the mammalian homologs [22], suggest that a substantial selective pressure may be active on the β2m gene.

The D76N mutation is a rather conservative substitution for a solvent exposed residue; nevertheless, it leads to a surprisingly drastic change in terms of thermodynamic stability, aggregation propensity of the protein and to systemic amyloidosis. Although the β2m D76N variant has been extensively characterized since its recent identification [4–6], the molecular and structural determinants of its peculiar properties remain elusive. In fact, both the high resolution crystal structure and the NMR analysis of the mutant did not reveal any significant structural features that could explain the D76N mutant striking properties [4, 5].

Here we sought to test whether the D76N instability and aggregation propensity could be related to the loss of one surface negative charge that would yield a higher pI mutant protein. To this aim, we designed an Asn-scanning mutagenesis study, and individually substituted all the aspartate residues (D34, D38, D53, D59, D96 and D98) of human β2m, other than D76. For each of the six single-site mutants, fold stability, aggregation propensity and structural rearrangements upon mutation were explored. The potential effects of such mutations on any partially folded or unfolded state, which cannot be ruled out, have not been investigated as falling beyond the main focus of this work.

The data obtained for the D-to-N mutants in solution show a substantial conservation of the molecular properties displayed by wt β2m. CD spectra indicate that all the D-to-N mutants display a virtually identical overall structural organization (Fig 2A). Thermal unfolding shows that all D-to-N mutations, other than D76N, are reflected by small variations of the protein Tm (up to 3.4°C) (Fig 2B). The aggregation propensities of all six D-to-N mutants resemble that of wt β2m, with no signs of the massive aggregation and amyloid formation reported for the D76N pathologic variant (Fig 3).

The crystal structures of D53N, D59N (this study) and of D76N [4] variants match closely that of wt β2m. Their overall 3D-structures are maintained without rearrangement of the loops or of the secondary structures; even locally, the mutations are quite well accepted, with minimal rearrangements of neighbouring side chains. Such a result is not surprising, as residues 53, 59 and 76 are fully solvent exposed, being located in the D-strand, the DE-loop and the EF-loop, respectively; in addition, flexibility characterizes the 59 and 76 mutation sites (Fig 6). Although detailed structural analysis is prevented for the D34N and D96N mutants that could not be crystallised, their CD spectra indicate a substantial conservation of the native β2m fold upon mutation (Fig 2). Moreover, the slopes of the unfolding curves suggest that the level of unfolding cooperativity is the same for all the variants.

Separate structural considerations should be drawn for the D38N and D98N variants. In both cases, the mutated Asp residues are surrounded by other charged residues (positive for D38, negative for D98). The D38N mutation leads to the rearrangement of several charged residues in the vicinity of the mutation site (Fig 5) as a result of newly established electrostatic and H-bond interactions. Although this mutant is the most destabilised (ΔTm of—3.4°C), the Tm drop is much lower than in the case of the D76N variant (ΔTm of—10.4°C) and, importantly, D38N does not display increased aggregation propensity. However, what is observed for the D38N mutant structure is in contrast with what has been reported for the D76N variant. As in the D38 case, residue D76 is surrounded by positively charged side chains, but the D-to-N mutation virtually does not trigger any rearrangement of their conformations.

Even if the D98N structure is well superimposable with that of wt β2m, the D98N mutant displays local increased flexibility in the C-*terminus* and the neighbouring EF-loop; such flexibility is somewhat unexpectedly mirrored by a slightly increased fold stability (ΔTm + 1.3°C). We can thus conclude that the removal of the D negative charge from the 38 and 98 sites, which are surrounded by heavily charged regions of the protein, leads to measurable structural effects.

The data here presented on the D-to-N mutations may be summarised as follows: (i) none of the engineered D-to-N β2m mutants matches the drastic effects in stability and in aggregation propensity observed for the pathologic D76N variant; (ii) none of the D-to-N mutations cause major conformational changes in the protein but, opposite to the D76N mutant, if the D-to-N mutation is located in a charge-rich region it leads to some structural rearrangements or increased flexibility; (iii) even if highly conserved through species, six out of the seven β2m D surface residues do not contribute substantially to the stability of monomeric β2m, as shown by the contained negative ΔTm values observed for D34N, D38N and D59N, and by the modest increased stability experienced by D96N and D98N.

Taken together, the results of our Asn-scan study indicate that random removal of a surface negative charge is not sufficient to produce the aggressive phenotype observed for the D76N variant. Thus, it is not the overall net charge of the protein (or its pI), the secondary structure location of the D-to-N mutation (in a β-strand or in a flexible loop), or the overall charge distribution on the β2m surface, that firstly control protein stability and aggregation propensity. The data here presented therefore indirectly support the idea that D76 occupies a unique site within the β2m fold, playing a specific role in its thermodynamic stability and aggregation trends. The phenotype produced by the substitution of D76 with an isosteric but neutral residue cannot be even partly matched through D-to-N mutations at other β2m sites, suggesting that, indeed, the removal of a surface negative charge may matter, but that the spatial location of the mutated residue in the context of the protein architecture is a non-negligible feature.
